# Dabska tumor (Endovascular papillary angioendothelioma) of testis: a case report with brief review of literature

**DOI:** 10.1186/1746-1596-1-12

**Published:** 2006-07-22

**Authors:** Alka Bhatia, Ritambra Nada, Yashwant Kumar, Prema Menon

**Affiliations:** 1 Department of Histopathology, Postgraduate Institute of Medical Education and Research, Chandigarh, India; 2 Department of Pediatric surgery, Postgraduate Institute of Medical Education and Research, Chandigarh, India

## Abstract

The Dabska tumor also known as Endovascular papillary angioendothelioma is a rare type of hemangioendothelioma characterized by intraluminal papillary endothelial structures. Most of these are superficial in location but occurrence in deeper tissues is also known. We describe case report of testicular Dabska tumor in a child presenting as inguinal hernia. To the best of our knowledge this is the first case report describing the occurrence of this rare entity in testis.

## Background

The Dabska tumor is a rare, low-grade angiosarcoma that often affects the skin and subcutaneous tissues of children [1]. Microscopically, it is characterized by anastomosing vascular channels, some of which contain papillary projections or tuft-like structures sometimes resembling renal glomeruli. These vascular channels as well as papillations are lined by cuboidal endothelial cells and are often flanked by dense hyalinized zones containing lymphocytes. The hyalinized material is thought to represent the basement membrane material possibly synthesized by tumor cells [2]. Although rare cases of Dabska tumors have been reported in deeper locations, there is no case report describing the occurrence of this entity in testis. We therefore report first case of Dabska tumor arising in testis of a 1 year, 10 month old child who first presented as inguinal hernia.

## Case report

A 1 year 10 month old male child was referred to our pediatric surgery department for pain and tenderness in a scrotal swelling for past 2 days. The swelling was diagnosed as non-obstructive inguinal hernia at the referral centre at 2 months of age. As it was not progressing the parents were advised to wait till the age of one year. There was no history of any trauma to genitalia. On examination the scrotum was swollen on left side and left testis was not palpable separately. The right testis was normally palpable in scrotal sac. Ultrasonographic examination of the swelling showed a markedly enlarged testis with a heterogenous echotexture. A possibility of testicular tumor was suggested and Fine Needle Aspiration Cytology (FNAC)/biopsy were advised. The FNAC showed few clusters of spindle cells and some cells with prominent nucleoli and vacuolated cytoplasm. However no definite opinion was given. A biopsy was also done which was reported as infarction of testis with extensive vascular proliferation. Subsequently a high left inguinal orchidectomy was carried out. Intraoperatively the hernial sac contained clear fluid and no bowel loops were seen. The specimen was sent for histopathology.

**Figure 1:**
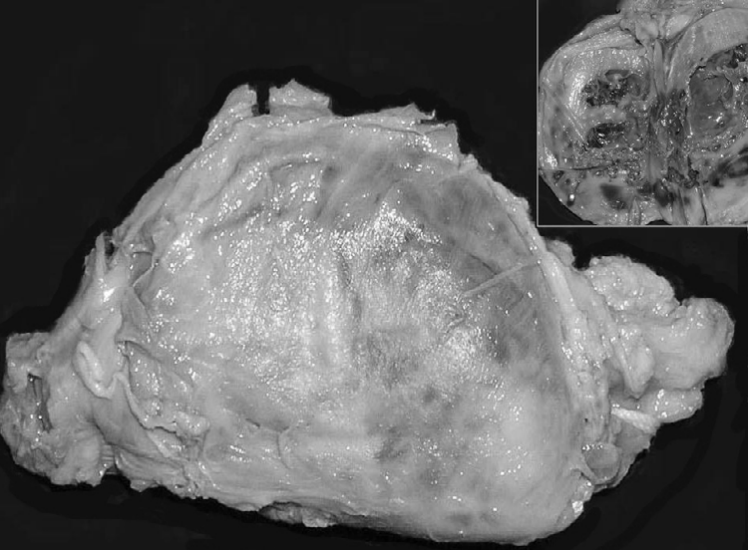
Gross photograph of left testis replaced by the tumor. The outer surface shows brownish discoloration. Note that the shape of testicle is maintained. Cut surface is solid-cystic with areas of hemorrhage (Inset).

**Figure 2:**
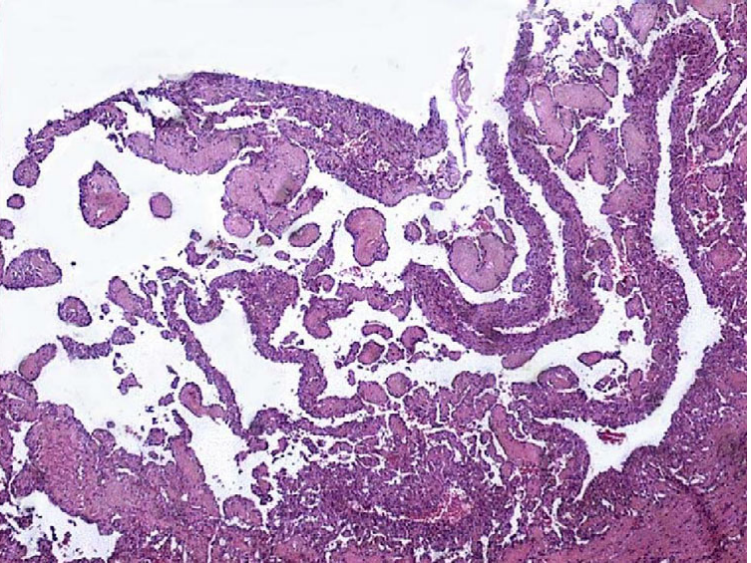
Low power photomicrograph of the tumor showing papillae with hyalinized cores.

**Figure 3:**
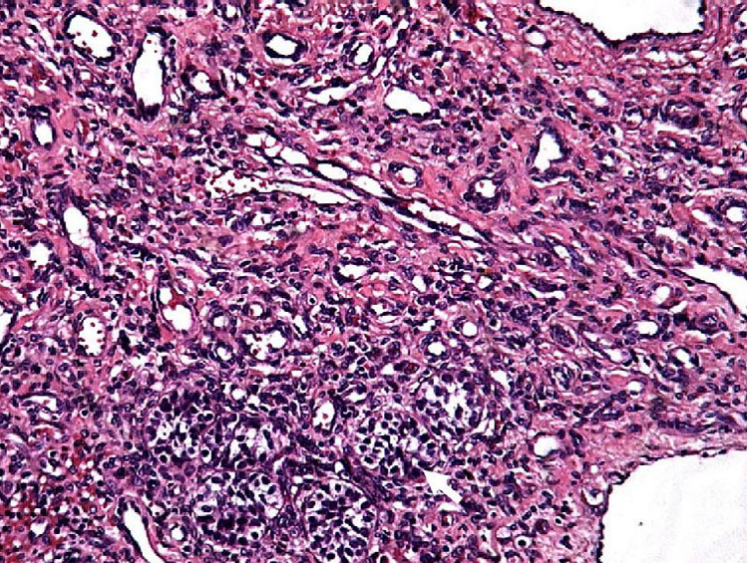
High power view showing a vascular tumor with cuboidal endothelium lining the vessels. Few entrapped seminiferous tubules are also noted (arrow).

The gross examination revealed a tumor replacing the entire testis; however shape of the testicle was maintained (figure 1). The tumor measured 4 cm in diameter. Outer surface was smooth and brownish in colour. The cut surface was also reddish brown with areas of hemorrhage. Though the tumor was solid, prominent cystic change and necrosis were seen in the central portion. Microscopic examination showed a vascular tumor composed of anastomosing, irregular vascular channels forming papillae in some areas. These papillae had hyalinized cores and were lined by atypical endothelial cells (figure 2). The vascular channels were also lined by plump cuboidal endothelial cells with focal hobnailed appearance and lymphocytic sprinkling. Frequent intracytoplasmic vacuolations and many mitotic figures were identified. The background showed large areas of hemorrhage and infarction. Few residual seminiferous tubules were found entrapped within the tumor tissue (figure 3). Immunohistochemically, the tumor cells showed strong positivity for CD34. The spermatic cord was normal.

## Discussion

The Dabska tumor was first described in 1969 by Maria Dabska in skin and subcutaneous tissues in children and named as malignant endovascular papillary angioendothelioma. She described 6 cases in her series [3]. Since then approximately 30 cases have been described in the literature. Of these 18 were children and 12 were adults. The age of the patients ranged from birth to 83 years and there was no sex predilection [4,5]. Besides skin and subcutaneous tissue, the tumor has been described in other deeper locations like spleen [6], soft tissues [7], bone [8], and tongue [9]. Generally it presents as a slow-growing, painless, usually intradermal nodule that grows to 2–3 cm in diameter. In our case this tumor presented as inguinal hernia and after surgery was found to be a solid mass with cystic change. This kind of presentation has not been described in any of the previous studies. At low power, Dabska tumor appears similar to cavernous lymphangiomas. The cuboidal or hobnail endothelial cells lining the vascular structures are characterized by a high nuclear cytoplasmic ratio and an apically placed nucleus that produces a surface bulge, accounting for the term "hobnail" or "matchstick" [1]. Individual endothelial cells range from cuboidal to tall and cylindrical with vacuolated cytoplasm and hyperchromatic eccentric nuclei on their luminal border. Mitotic figures may be seen, as in our case. Many intraluminal lymphocytes may also be evident, often attached to the endothelial cells. All these features were noted in our case. Immunohistochemically the tumor cells are positive for Von-Willebrand factor, CD31, CD34 and vascular endothelial growth factor receptor-3 (VEGFR-3). The staining for CD34 and VEGFR-3 is most intense out of these [1]. In the present case strong positivity for CD34 immunostaining was observed in tumor cells. Ultrastructural examination demonstrates tumor cells with irregular nuclei, abundant perinuclear cytoplasmic filaments, and many pinocytotic vesicles. Weibel-Palade bodies may be evident. The hyaline globules consist of electron-dense basement membrane material [6].

Along with a closely related tumor, the retiform hemangioendothelioma it comes under the category of Hobnail hemangioendothelioma, a subtype of hemangioendothelioma. Pathophysiologcally it has been viewed as a distinct intralymphatic neoplasia and renamed as papillary intralymphatic angioendothelioma [10]. These tumors are known to occur in previously existing benign vascular lesions like cavernous hemangiomas [7]. In our case no cavernous hemangioma like areas could be found even after extensive sampling

Orchidectomy is the treatment of choice. Prognostically these tumors are low grade lesions with a capacity to extend to regional lymph nodes [1]. Three of the original 6 cases were locally aggressive, with tumor invasion into deeper structures, including bone, musculature, fascia, and/or tendons. One of Dabska's original 6 Dabska tumor patients ultimately died of widespread pulmonary metastases [11]. Therefore, Dabska tumor though believed to have a favorable prognosis, can be locally invasive with a potential to metastasize. In the present case there was no evidence of any nodal involvement or distant metastasis and the child was doing well on last follow up after 1 year of surgery.

Our case describes the first occurrence of this entity in testis with an unusual presentation in the form of inguinal hernia. It also highlights the difficulty in diagnosing such tumors on FNAC or small biopsy specimens as it was mistaken for an infarct on biopsy.
